# Optimizing Ultrasound-Assisted Deep Eutectic Solvent Extraction of Bioactive Compounds from Chinese Wild Rice

**DOI:** 10.3390/molecules24152718

**Published:** 2019-07-26

**Authors:** Jia Zeng, Yuqing Dou, Ning Yan, Na Li, Huaibao Zhang, Jia-Neng Tan

**Affiliations:** 1 Tobacco Research Institute of Chinese Academy of Agricultural Sciences, Qingdao 266101, China; 2 Graduate School of Chinese Academy of Agricultural Sciences, Beijing 100081, China; 3 Key Laboratory of Oilseeds Processing, Ministry of Agriculture, Wuhan 430062, China

**Keywords:** deep eutectic solvents, Chinese wild rice, bioactive compounds, response surface methodology, UPLC-QqQ-MS

## Abstract

In this study, deep eutectic solvents (DESs) were used for the ultrasound-assisted extraction (UAE) of valuable bioactive compounds from Chinese wild rice (*Zizania* spp.). To this end, 7 different choline chloride (CC)-based DESs were tested as green extraction solvents. Choline chloride/1,4-butanediol (DES-2) exhibited the best extraction efficiency in terms of parameters such as the total flavonoid content (TFC), total phenolic content (TPC), and free radical scavenging capacity (DPPH^●^ and ABTS^●+^). Subsequently, the UAE procedure using 76.6% DES-2 was also optimized: An extraction temperature of 51.2 °C and a solid–liquid ratio of 37.0 mg/mL were considered optimal by a Box–Behnken experiment. The optimized extraction procedure proved efficient for the extraction of 9 phenolic and 3 flavonoid compounds from Chinese wild rice as determined by quantification based on ultra-performance liquid chromatography–triple quadrupole tandem mass spectrometry (UPLC-QqQ-MS). This work, thus, demonstrates the possibility of customizing green solvents that offer greater extraction capacity than that of organic solvents.

## 1. Introduction

Chinese wild rice (*Zizania latifolia*), a nutritious whole grain, has been consumed in China for at least 3,500 years. It contains proteins; minerals; vitamins; and a variety of phytochemicals such as phenolics, saponins, phytosterols, and anthocyanins [1,2,3], which can be attributed to many bioactivities including antioxidant, anti-inflammatory, antiobesity, and antianaphylactic actions [4,5,6,7,8]. However, phenolic acids and flavonoids obtained from Chinese wild rice that demonstrates significant antioxidant activity have not been fully investigated and utilized [9]. The total phenolic content (TPC) of *Z. latifolia* varies from 1698 to 4108 μg/g, with ferulic, sinapic, and vanillic acids being the main compounds [4], which are responsible for a wide range of bioactivities, including antioxidant, antiallergenic, and antimicrobial effects [5,10]. The total flavonoid content (TFC) of *Z. latifolia* varies from 578 to 791 μg/g and mainly comprises procyanidin, quercetin, and catechin [4], which improve insulin sensitivity and blood glucose homeostasis, inhibit proliferation of tumor cells, and demonstrate antioxidant activity [6,7,11,12,13]. 

Therefore, the characterization and quantification of phenolic compounds from Chinese wild rice (*Zizania* spp.) is a task of high practical significance. However, these active substances need to be extracted prior to their use. The traditional extraction processes using organic solvents often induces toxicity and involves the use of flammable, explosive, and poorly biodegradable substances. Therefore, it is necessary to find a green extraction technology for such purposes [14,15,16,17,18].

Because green extraction offers optimal utilization of solvents, minimizes environmental impact of the process, and benefits human health, it is highly desired in academic and industrial research for the development of effective extraction processes [19,20,21,22]. Because deep eutectic solvents (DESs) are inexpensive, easy to prepare, and mostly biodegradable and show extremely low toxicity, they have attracted widespread attention [23,24,25,26]. A DES usually consists of two cheap and safe components: hydrogen bond receptors (usually choline chloride) and hydrogen bond donors (glycerol, sugar, alcohol, etc.). The melting point of the eutectic mixture formed by these components is lower than that of each individual component [16,27,28]. An increasing number of studies have investigated the extraction of bioactive plant compounds, including flavonoids and phenolic acids, using DESs as extraction media [14,15,16,17,18,21,24,25,26,27,28,29,30,31,32]. However, no reports have been made on the use of DESs to extract phenolic compounds from Chinese wild rice, on a comprehensive comparison of the results obtained using different DESs, and on the quantitative detection of phenolic compounds.

In this study, we achieved the simultaneous extraction and quantification of 9 phenolic and 3 flavonoid compounds from Chinese wild rice using DES and UPLC-MS. The extraction efficiencies of 7 DESs were compared to select the most suitable solvent for further experiments. After the optimization of reaction conditions, the extract was quantitatively analyzed by UPLC-QqQ-MS.

## 2. Results and Discussion

### 2.1. Evaluation of DES Extraction Efficiency

A good biodegradable, low-cost hydrogen bond acceptor, ChCl, and a series of hydrogen bond donor compounds produce environmentally friendly DESs. Herein, combining ChCl and four types of hydrogen bond donors—alcohol (glycerol, 1,4-butanediol), sugar (D-fructose, D-glucose), carboxylic acid (lactic acid, DL-malic acid), and amine (urea)—produced seven DESs (Table 4). All extraction processes conformed to the following conditions: extraction time of 20 min, extraction temperature of 50°C, and solid–liquid ratio of 50 mg/mL.

**Figure 1:**
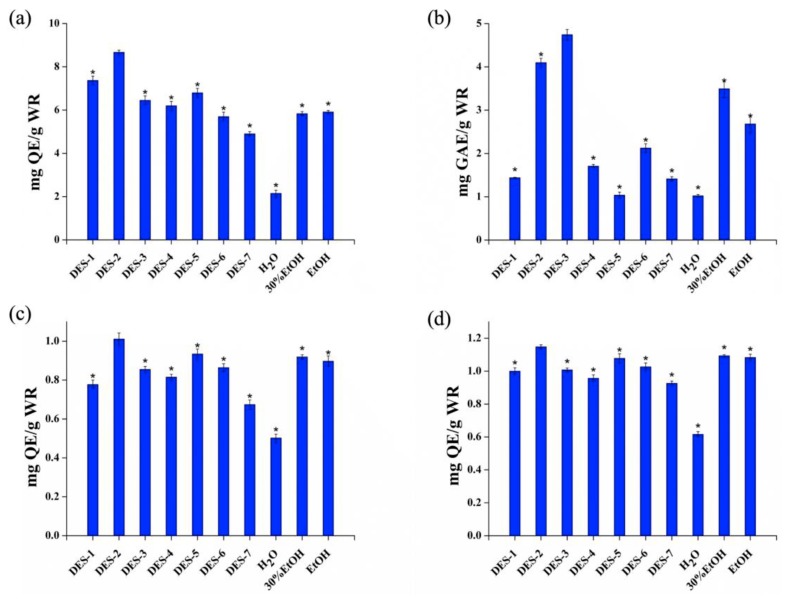
Extraction efficiency of 7 deep eutectic solvents (DESs) and three conventional solvents: (**a**) total flavonoid content (TFC); (**b**) total phenolic acid content (TPC); (**c**) radical scavenging capacity determined by DPPH^●^ assays; and (**d**) radical scavenging capacity determined by ABTS^●+^ assays. Extraction efficiencies that differed significantly from those of DES-2 are indicated with an asterisk * (*p* < 0.05). Data were analyzed with ANOVA followed by post hoc Tukey’s test.

The DES extraction efficiency was evaluated by considering the TFC and TPC of the corresponding extract. As representative bioactive phenolic compounds, quercetin and gallic acid were used to quantify the TFC and TPC, respectively. Furthermore, two radical scavenging capacities (DPPH^●^ and ABTS^●+^) were measured simultaneously. The wild rice extract prepared using 7 different DESs was studied in comparison with those obtained using pure water and the conventional solvents 30% EtOH and EtOH. The obtained TFC values are compared in Figure 1a, which shows that the performance of DES-2 (8.67 ± 0.15) exceeded those of DES-1 (7.37 ± 0.2), DES-3 (6.45 ± 0.19), DES-4 (6.2 ± 0.15), DES-5 (6.8 ± 0.2), DES-6 (5.7 ± 0.17), DES-7 (4.9 ± 0.1), H_2_O (2.15 ± 0.06), 30% EtOH (5.83 ± 0.13), and EtOH (5.91 ± 0.07) and that the difference was significant. The TFC extracted from DES-2 was slightly lower than the TFC reported by Chu et al. [9]. However, our novel strategy may represent a more sustainable approach for the extraction and separation of natural products. More traditional solvents can be deposited to make their phytochemistry more attractive and environmentally friendly. Figure 1b compares the obtained TPC values. Unexpectedly, DES-3 (4.74 ± 0.12) showed the best results of all tested solvents, followed by DES-2 (4.10 ± 0.10) which were higher than those of native solvents [9]. A recent study pointed out that almost all tested ChCl-based DESs showed better extraction yields for the phenolic compounds from mulberry leaves. Compared with the traditional solvents (water or MeOH solution), the tailor-made ChCl/citric acid proved to be an excellent extraction solvent for a broad range of phenolic compounds with various polarities [33]. In the experimental assay, TFC values were highly correlated with DPPH^●^ (R = 0.851) and ABTS^●+^ (R = 0.871) (Figure S2). Therefore, DES-2 was used for subsequent experiments.

### 2.2. Optimization of Extraction Condition

**Figure 2:**
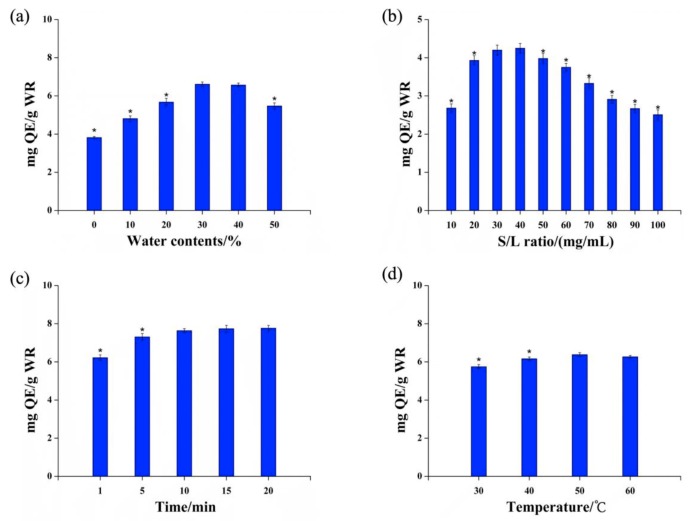
Effect of (**a**) water content in DES, (**b**) solid–liquid ratio, (**c**) extraction temperature, and (**d**) extraction time on the extraction yield.

Using the best-performing DES (choline chloride/1,4-Butanediol, i.e., DES-2), the effect of extraction conditions (DES moisture content, extraction temperature, extraction time, and solid–liquid ratio) on the TFC were further investigated (Figure 2).

#### 2.2.1. DES Water Content

When the extraction temperature was 50 °C, the extraction time was 20 min and the solid–liquid ratio was 50 mg/mL; the TFC first rose and then declined as the moisture content of DES increased from 0% to 50%. The TFC reached its highest value at 30% moisture content. Therefore, a better DES moisture content of 30% was selected and the TFC was (6.61 ± 0.01) mg/g at this value.

#### 2.2.2. Extraction Temperature

Under the extraction conditions of a DES moisture content of 30%, an extraction time of 20 min, and a solid–liquid ratio of 50 mg/mL, when the extraction temperature increased within the range of 30–50 °C, the TFC tended to rise and the extraction yield reached the maximum value at 50 °C. After that, the TFC decreased slightly with increases in the temperature, but the difference was not significant (P > 0.05). Therefore, the extraction temperature of 50 °C was selected as optimal, at which the TFC was (6.38 ± 0.10) mg/g.

#### 2.2.3. Extraction Time

Under the extraction conditions with a DES moisture content of 30%, an extraction temperature of 50 °C, and a solid–liquid ratio of 50 mg/mL, when the extraction time increased within the range of 1–10 min, the TFC exhibited an upward trend and the extraction yield reached the maximum value at 10 min. After that, the extraction yield increased slightly with time, but the difference was not significant (P > 0.05). In terms of saving time and improving reaction yield, the preferred extraction time was thus 10 min, at which the TFC was 7.64 ± 0.10 mg/g.

#### 2.2.4. Solid–Liquid Ratio

Under the extraction conditions of a DES water content of 30%, an extraction temperature of 50 °C, and an extraction time of 20 min, when the solid–liquid ratio increased in the range of 40–100 mg/mL, the TFC showed a downward trend. While the solid–liquid ratio was 10–40 mg/mL, the TFC increased. In terms of saving solvent and controlling the extraction cost, the optimal solid–liquid ratio was selected to be 40 mg/mL and the TFC at this value was (4.25 ± 0.13) mg/g.

### 2.3. Optimization of Experimental Design Conditions

The Box–Behnken response surface test was designed based on single factor experiments. The extraction efficiency of total flavonoids from wild rice was effectively optimized against the DES water content (variable A), extraction temperature (variable B), and solid–liquid ratio (variable C), with each of the three levels considered as independent variables. In order to effectively evaluate the extraction efficiency, the TFC was responded. To avoid systematic errors, all experiments were performed in a random order. The response surface factor coding and corresponding variable levels are listed in Table 1. A second-order polynomial equation was used to perform multiple regression analysis on the experimental data and to establish a model. The regression model equations based on the response and variables of the coding level are as follows:

Y = 9.01 − 0.42A + 0.19B − 0.63C − 0.22AB + 0.35AC + 0.060BC − 1.11A^2^ − 0.28B^2^ − 0.55C^2^

where A is the DES water content, B is the extraction temperature, and C is the solid–liquid ratio.

The results of the model regression analysis of variance are summarized in Table 2. It can be seen that the regression model P = 0.0006, indicating that the regression model reached an extremely significant level and that the equation determination coefficient R^2^ = 0.9567, indicating that the equation showed a high degree of fit. The lack of fit F = 0.41, P = 0.1453, was not significant. Therefore, the model can be used to analyze and predict total flavonoids in wild rice.

**Figure 3:**
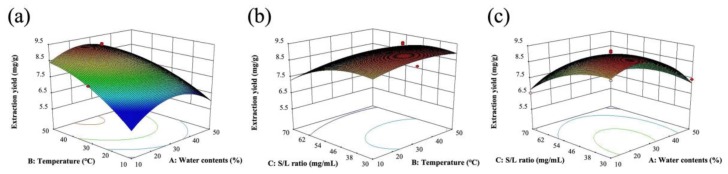
Response surfaces of total flavonoid content (TFC) for optimization: (**a**) deep eutectic solvent (DES) water content, (**b**) extraction temperature, and (**c**) solid–liquid ratio.

For graphical interpretation of the significant effects of interactions among the three variables on the TFC, we used a three-dimensional (3-D) response surface plot of the model (Figure 3), which shows that the extraction yield of TFC is apparently related to the main variable. The DES moisture content (variable A) and solid–liquid ratio (variable C) were statistically extremely significant, indicating that the DES moisture content and the solid–liquid ratio have an extremely significant effect on the extraction efficiency. In contrast, the extraction temperature (B) showed no significant effect (P > 0.05).

On solving the equation by software Design Expert 8.0.5, the optimal extraction conditions were determined to be as follows: DES moisture content of 23.4% (w/w), extraction temperature of 51.2 °C, and a solid–liquid ratio of 37.0 mg/mL. Under these conditions, the TFC extraction yield predicted by the response surface model was 9.38 mg/g. In order to test the reliability of the model, a three-dimensional verification test was carried out using the optimized process conditions. The average extraction yield of the TFC of the wild rice was 9.30 mg/g, and compared with the theoretical prediction, the relative deviation was 1.04%. It is thus demonstrated that the equation is essentially applicable to actual cases, and the correctness of the response surface model was verified, which can be used for the theoretical prediction of the TFC extraction yield in wild rice.

### 2.4. Quantification of Antioxidants

The contents of compounds in the DES crude extract acquired from wild rice and control samples were determined using the multi-reaction monitoring (MRM) mode of UPLC-QqQ-MS/MS. Due to the lack of available standards, only 9 phenolic and 3 flavonoid compounds were quantified.

In terms of the phenolic acid profile of wild rice, high contents of vanillin, *p*-hydroxybenzaldehyde, *p*-hydroxybenzoic acid, protocatechuic acid, ferulic acid, and sinapic acid were found. As shown in Table 3, ferulic acid was the most abundant phenolic acid in all crude extracts, which is in agreement with the results reported by Sumczynski et al. [4]. The highest concentration of ferulic acid, found in the DES-2 crude extract of wild rice, was 114.84 μg/g. Sinapic acid showed the second highest amount, ranging from 42.00 to 103.50 μg/g. Among them, the contents of DES-2 and 3 were higher than that of traditional solvents used in methanol extraction (25.4 and 53.6 μg/g). Beside ferulic and sinapic acid, vanillin, *p*-hydroxybenzaldehyde, and vanillic acid were also found in the DES-6 (18.01 μg/g), DES-6 (18.82 μg/g), and DES-3 (17.04 μg/g) extracts, respectively, in significant quantities (P < 0.05). The contents of vanillin and *p*-hydroxybenzaldehyde in the crude extract of DES-6 were higher than that of native solvents (13.1 and 15.6 μg/g) [9]. In addition, the DES-6 and DES-7 crude extracts of wild rice were rich in *p*-coumaric acid and protocatechuic acid. The study established the following order of total phenolic acids in the DES crude extract of wild rice: DES-2 (267.59 μg/g) > DES-3 (247.55 μg/g) > DES-6 (235.59 μg/g) > DES-1 (224.65 μg/g) > DES-7 (219.15 μg/g) > DES-5 (161.89 μg/g) > DES-4 (103.48 μg/g). The study of Ozturk et al. [34] also showed that the ChCl-based DESs paired with glycerol and ethylene glycol have been proved to outperform conventional solvents (aq. ethanol 30 wt.% water) in the extraction of polyphenolic compounds from orange peel, both in Terms of TPC and antioxidant activity of the extracts. They found that the structural analysis of biomass before and after extraction suggested DES as an efficient solvent for cell wall dissolution, which can be related with their higher hydrogen bond basicity that allows efficient intermolecular interactions between the solvent and the cellulose strands, which may be the reason for the relatively high efficiency of DES extraction. 

As for flavonoids, only catechin, procyanidin B1, and quercetin were detected in wild rice. The highest values of catechin and quercetin were assessed in the DES-2 (23.51 and 21.12 μg/g, respectively) extract. At the same time, the contents of catechin and quercetin extracted by DES-2 were also higher than that by methanol extraction (22.1 and 11.4 μg/g, repectively) [4]. In addition, the content of procyanidin B1 was the highest in DES-4 (12.87 μg/g). However, compared with the study of Chu et al. (13.0 μg/g) [9], we obtained a slightly lower content of procyanidin B1. Despite this, DESs have an irreplaceable advantage over traditional solvents, such as easy preparation and extremely low toxicity, because of which DESs are widely used to extract bioactive plant compounds.

As future research, considering the beneficial effect of DESs on the extraction efficiency of target compounds, extraction by using developed DESs can be studied to be an alternative to green and efficient extraction of phenolic compounds from natural sources.

## 3. Materials and Methods

### 3.1. Materials, Reagents, and Equipment

The Chinese wild rice sample was obtained from the Hubei province, China; was ground using a pulverizer; and was dried at 40 °C until the water evaporated completely. The resulting powdered sample was stored in the dark at –20 °C.

The reagents used in the synthesis of DES—choline chloride (≥98%), glycerol (≥99.0%), 1,4-butanediol (≥99.0%), D-fructose (≥99.0%), D-glucose (≥99.0%), lactic acid (≥85.0%), malic acid (≥99.0%), urea (≥99.0%), and ethanol (≥99.0%)—were all obtained from Sinopharm Chemical Reagent Co., Ltd (Shanghai, China). Deionized water was prepared using a Milli-Q® Ultrapure Water System (Millipore, Billerica, MA, USA).

Analytical standards including vanillin (≥97%), *p*-hydroxybenzaldehyde (≥97%), *p*-hydroxybenzoic acid (≥97%), *p*-coumaric acid (≥98%), protocatechuic acid (≥98%), syringic acid (≥ 98%), ferulic acid (≥98%), sinapic acid (≥98%), vanillic acid (≥97%), procyanidin B1 (≥97%), and catechin (≥97%) were obtained from Beijing Solarbio Technology Co., Ltd. (Beijing, China).

Reagents for the determination of TFC, TPC, DPPH^●^, and ABTS^●+^, including the Folin reagent and gallic acid, were purchased from Sigma-Aldrich (St. Louis, MO, USA). Aluminum chloride (≥99.0%) was purchased from Aladdin Industrial Corporation (Shanghai, China); ABTS (≥98.0%), quercetin (≥98.0%), and DPPH (≥96.0%) were obtained from Shanghai Macklin Biochemical Co., Ltd (Shanghai, China); and sodium nitrite (≥98.5%), sodium carbonate (≥99.8%), methanol (≥99.5%), sodium hydroxide (≥96.0%), and potassium persulfate (≥99.5%) were purchased from Sinopharm Chemical Reagent Co., Ltd (Shanghai, China).

Chromatographic-grade acetonitrile was purchased from Merck Chemical Technology (Shanghai) Co., Ltd. (Shanghai, China), and acetic acid (≥ 99.8%) was obtained from Sinopharm Chemical Reagent Co., Ltd (Shanghai, China).

An Acquity UPLC system (Waters Corp., Milford, MA, USA) coupled with a TSQ Quantum triple quadrupole tandem mass spectrometry instrument (Thermo Scientific, San Jose, CA, USA) was used. The magnetic stirrer MS-H-Pro+ was obtained from Shanghai kehuai Instrument Co., Ltd. (Jiangsu, China). The Vortex, Lab dancer was obtained from IKA (Guangzhou) Instrument Equipment Co., Ltd. (Guangzhou, China). The ultrasonic water bath SBL-30DT was obtained from SCIENTZ Biotechnology Co., Ltd. (Ningbo, China). The centrifuge TDZ5-WS was obtained from Changsha High-tech Industrial Development Zone Xiangyi Centrifuge Instrument Co., Ltd. (Changsha, China). The centrifuge tube was obtained from Beijing Labgic Technology Co., Ltd. (Beijing, China). The microplate reader Multiskan^TM^ FC was available from Thermo Fisher Scientific (China) Co., Ltd. (Shanghai, China). The 96-well plate costar 3599 was available from Thermo Fisher Scientific (China) Co., Ltd. (Shanghai, China).

### 3.2. DES Preparation

The DESs used in the study were prepared by the method described [24,35]. The desired components were added to a round-bottomed flask in the required molar ratio, and a magnet was introduced, following which the mixture was subjected to magnetic stirring at 80°C until a stable, uniform, transparent liquid formed in the bottle. The seven DESs synthesized are listed in Table 4. In order to facilitate the experimental operation, 30% moisture was added to each DES.

### 3.3. Ultrasonic Extraction of Wild Rice Powder

In this study, we used ultrasound-assisted extraction to extract flavonoids and phenolic compounds, determined the TFC and TPC from wild rice powder with DES, and tested them for oxidation resistance. First, we weighed 50 mg of wild rice powder in a 10-mL glass tube and then accurately and quickly added 2 mL of DES in a glass tube, placed the mixture on a vortex meter, and stirred it well (10 s). The ultrasonic water bath settings were set in advance at 20 min, 50 °C, 25 kHz, and 200 W. At the end of stirring, a solid–liquid mixture was obtained. After the tube cooled to room temperature, it was placed in a centrifuge and centrifuged at 3000 rpm for 10 min. The supernatant (1 mL) was diluted with methanol (4 mL), and the mixture was passed through a 0.22-μm filter for subsequent analysis. Each extraction was performed in triplicate, and the extraction yield (E_y_) was calculated as follows:

E_y_ = (C_0_ × V_0_)/M_0_

where C_0_ is the concentration of phenolic acid or flavonoid found in DES by UPLC-MS analysis, V_0_ is the volume of the diluted liquid, and M_0_ is the mass of the sample.

### 3.4. Extraction Efficiency Evaluation

#### 3.4.1. Quantification of Nine Phenolic Acids and Three Flavonoids

Vanillin, *p*-hydroxybenzaldehyde, *p*-hydroxybenzoic acid, *p*-coumaric acid, protocatechuic acid, syringic acid, ferulic acid, sinapic acid, vanillic acid, proanthocyanidin B1, catechin, and quercetin were all quantitatively analyzed by ultra-performance liquid chromatography–triple quadrupole tandem mass spectrometry (UPLC-QqQ-MS/MS) using an Acquity UPLC system (Waters Corp., Milford, MA, USA) coupled with a TSQ Quantum triple quadrupole tandem mass spectrometer (Thermo Scientific, San Jose, CA, USA). For gradient elution, we used solvent A (0.1% acetic acid in acetonitrile, v/v) and solvent B (0.1% acetic acid in H_2_O solution, v/v) under the following conditions: column, 2.1 mm × 50 mm; 1.7 μm C18 particles; injection volume, 1 μL; flow rate, 0.3 mL/min; column temperature, 25 °C; and gradient procedure as follows: 0–5 min, 5–10% A; 5–7 min, 10–20% A; 7–8 min, 20–60% A; 8–9 min, 60–100% A; 9–10 min, 100–5% A; and 10–12min, 95% A.

An electrospray ionization (ESI) source was used in the negative ion multiple reaction monitoring (MRM) mode. The optimized ion spray voltage was 3000 V. The vaporizer and capillary temperatures were 350 °C and 225 °C, respectively. Nitrogen was used as the sheath gas (30 arb) and auxiliary gas (5 arb), and argon was used as collision gas (1.5 mTorr). The collision energy was optimized individually for each transition. Data acquisition and processing were performed using the Xcalibur 3.1 software (Thermo Scientific, San Jose, CA, USA). The ion transitions and optimized MS parameters of the 12 external standards are listed in Table 5. The parameters of linear regression, concentration range, limits of detection (LOD), and limits of quantification (LOQ) for the analyzed standards are listed in supplemental Table S1. The UPLC-MS/MS TIC spectra of the standards are shown in supplemental Figure S1.

#### 3.4.2. Determination of TFC Content

The TFC measurement method used here followed an established procedure with some modifications [36]. Fifty microliters of a methanol blank control, a quercetin standard solution, and a 5-fold-diluted DES extract sample were thoroughly mixed with 10 μL of 5% NaNO_2_ in a 96-well plate. After 5 min, 10 μL of 10% AlCl_3_ was added and mixed well. After 1 min, 100 μL of NaOH was quickly added and mixed well. The absorbance values at 510 nm were immediately measured in a microplate reader, and each treatment was repeated 3 times. The quercetin concentration–absorbance value was used as a standard, and the TFC content was expressed in milligrams of quercetin equivalent per gram of wild rice (mg QE/g WR). The calibration curve for determining the quercetin content followed the equation y = 1.4156x + 0.0357 (r^2^ = 0.999, n = 6), and the concentration range was 10–100 μg/mL.

#### 3.4.3. Determination of TPC Content

The TPC measurement method was in accordance with the technique reported by Singleton et al. [37]. The sample reaction was carried out in a 5-mL plastic centrifuge tube. The Folin reagent was diluted 10 times with deionized water. Two hundred and fifty microliters of the diluted Folin reagent was added to the centrifuge tube, and then 250 μL of the methanol blank control, a gallic acid standard solution, and a 5-times-diluted DES extract sample was added to the centrifuge tube and vortexed at room temperature for 5 min for homogenization. Then, 1 mL of deionized water and 250 μL of a 20% Na_2_CO_3_ solution were added, after which the mixture was reacted in the dark for 30 min. The tube was placed in a centrifuge and centrifuged at 3000 rpm for 10 min. Finally, the reaction supernatant was aspirated into a 96-well plate and the absorbance of the reaction solution was measured at 740 nm: This measurement was repeated three times for each treatment. The gallic acid concentration–absorbance value was used as the standard, and the TPC content was expressed in milligrams of gallic acid equivalent per gram of wild rice (mg GAE/g WR). The calibration curve for determining the gallic acid content followed the equation y = 0.7449x + 0.1067(r^2^ = 0.9995, n = 6), and the concentration range was 100–1000 μg/mL.

#### 3.4.4. Determination of Radical Scavenging Capacity

The radical scavenging capacity was determined by the DPPH^●^ and the ABTS^●+^ assays. The antioxidant activity of the DES–wild rice extract was expressed in milligrams of quercetin equivalent per gram of wild rice (mg QE/g WR), and the two assays were used to establish a calibration curve equation for quercetin.

##### Determination of DPPH^●^ Content

DPPH^●^ scavenging capacity was measured as previously described [38]. A total of 50 μL of the DES extract and 150 μL of a 0.3 mM DPPH in methanol solution were added to a 96-well plate, mixed, and placed in the dark at room temperature for 30 min. Each treatment was repeated four times, and the absorbance was measured at a wavelength of 510 nm using the microplate reader. The DPPH^●^ free radical scavenging rate was calculated according to the following equation:

Clearance rate A (%) = (A_0_ − A_i_/A_0_) × 100

where A_0_ is the absorbance of the blank control and A_i_ is the absorbance of the sample reaction solution. Antioxidant activity curves were plotted based on the quercetin concentration. The calibration curve for determining the quercetin content followed the equation y = 37.456ln(x) + 183.19 (r^2^ = 0.997, n = 6), and the concentration range was 10–100 μg/mL.

##### Determination of ABTS^●+^ Content

The ABTS•^+^ radical was measured as previously described [39]. First, an ABTS^●+^ radical working solution was prepared and 1.1 mg/mL of ABTS in methanol solution was mixed with 0.68 mg/mL of a potassium persulfate aqueous solution in equal volumes; the mixture was allowed to stand overnight in the dark. Next, the absorbance value was adjusted to about 0.7, and 150 μL of the ABTS^●+^ free radical working solution and 50 μL of the DES extract were added to a 96-well plate, mixed, and reacted in the dark at room temperature for 30 min. Each treatment was repeated four times, and the absorbance values were measured at a wavelength of 734 nm using the microplate reader. The ABTS^●+^ free radical scavenging rate was calculated according to the following equation:

Clearance rate A (%) = (A_0_ − A_i_/A_0_) × 100

where A_0_ is the absorbance of the blank control and A_i_ is the absorbance of the sample reaction solution. The activity curve was plotted based on the concentration of quercetin. The calibration curve for determining the quercetin content followed the equation y = 34.855ln(x) + 178.63 (r^2^ = 0.9923, n = 6), and the concentration range was 10–100 μg/mL.

### 3.5. Experimental Design and Statistical Analysis

A single factor experiment was carried out for the yield of TFC from wild rice powder. The effects of the moisture content in the DES (0–50%), temperature (30–60 °C), solid–liquid ratio (10–100 mg/mL), and extraction time (1–20 min) on the TFC yield were investigated. The TFC was expressed in milligrams of quercetin equivalent per gram of wild rice (mg QE/g WR).

Based on the results of the single factor test, three factors with greater influence were selected according to the single factor analysis of the variance: water content (A, %), temperature (B, °C), and solid–liquid ratio (C, mg/mL). The Box–Behnken (BBD) model of the response surface analysis software was used to analyze the response surface of the TFC yield to determine the optimal process conditions for the TFC extraction. A total of 17 experiments were designed, including 5 center points. The response surface factor coding and respective variable levels are shown in Table 6. Data were processed using the Design-Expert 8.5 statistical software.

Statistical comparisons were made using the Single factor analysis of variance (ANOVA); P-values < 0.05 were considered significant. The Duncan significance test was performed using SPSS Statistics 17.0, with P-values < 0.05 being considered significant. Pearson correlation methods were used to assess the correlation between variables, with P-values < 0.01 being extremely significant. The data in the text were expressed as mean ± standard error (SD).

## 4. Conclusions

The factors that influence the performance of DESs and ultrasound-assisted extraction (UAE) were determined and optimized for the extraction of flavonoids and phenolic acids from Chinese wild rice. Among the DESs examined, 76.64% choline chloride/1,4-butanediol was observed to be the most promising extraction solvent: It was more effective than the other DESs and investigated conventional organic solvents. This DES can be considered reliable and efficient for the extraction of multiple phenolic and flavonoid analytes from Chinese wild rice, as confirmed by validation experiments. The following are the recommended optimized conditions for the UAE: an extraction temperature of 51.2 °C and solid–liquid ratio of 37.0 mg/mL. Also, 76.6% choline chloride/1,4-butanediol DES can also be used as a dilution solvent prior to UPLC analysis. Thus, the method proposed herein, based on UAE using DESs, provides a possible pathway to the green extraction of bioactive compounds from plant materials, particularly those showing potential for biochemical and pharmaceutical applications.

## Figures and Tables

**Table 1:** Response surface optimization experiments using DES extraction of investigated variables.

	Experiment Design						Response
No.	Coded Variables			Variables			Extraction Yield (mg/g)
	A	B	C	A (%)	B (°C)	C (mg/mL)	
1	0	−1	−1	30	30	30	8.75
2	0	0	0	30	45	50	9.10
3	−1	−1	0	10	30	50	7.62
4	0	0	0	30	45	50	8.90
5	0	0	0	30	45	50	9.15
6	0	−1	1	30	30	70	7.52
7	1	0	−1	50	45	30	7.42
8	1	−1	0	50	30	50	6.93
9	0	1	1	30	60	70	7.72
10	0	0	0	30	45	50	8.70
11	−1	0	−1	10	45	30	8.65
12	1	0	1	50	45	70	6.74
13	−1	0	1	10	45	70	6.56
14	−1	1	0	10	60	50	8.76
15	1	1	0	50	60	50	7.16
16	0	1	−1	30	60	30	8.72
17	0	0	0	30	45	50	9.20

**Table 2:** Analysis of variance for regression model equation.

Source	Sum of Squares	df	Mean Square	F Value	P-Value Prob > F
Model	12.90	9	1.43	17.19	0.0006
A	1.39	1	1.39	16.70	0.0047
B	0.30	1	0.30	3.59	0.1001
C	3.13	1	3.13	37.54	0.0005
AB	0.20	1	0.20	2.41	0.1644
AC	0.50	1	0.50	5.99	0.0443
BC	0.014	1	0.014	0.17	0.6923
A^2^	5.21	1	5.21	62.46	< 0.0001
B^2^	0.33	1	0.33	3.94	0.0876
C^2^	1.28	1	1.28	15.40	0.0057
Residual	0.58	7	0.083		
Lack of fit	0.41	3	0.14	3.20	0.1453
Pure error	0.17	4	0.043		
R^2^	0.9567				

**Table 3:** Quantitative determination of phenolic substances and control samples obtained from wild rice using various solvents: The extraction conditions were 30% of water in DESs; extraction temperature at 50°C; extraction time of 10 min, and solid–liquid ratio of 40 mg/mL. The results are expressed as μg/g of wild rice dry weight (n = 3).

Compound	DES-1	DES-2	DES-3	DES-4	DES-5	DES-6	DES-7	H_2_O	30%EtOH	EtOH
Flavonoids										
Catechin	12.87 ±0.1^e^	23.51 ±0.6^a^	11.64 ±0.1^f^	10.25 ±0.1^h^	15.99 ±0.1^c^	12.94 ±0.1^d^	nd	nd	17.90 ±0.1^b^	10.53 ±0.1^g^
Procyanidin B1	11.68 ±0.2^d^	10.02 ±0.3^f^	8.30 ±0.2^h^	12.87 ±0.2^a^	10.11 ±0.2^e^	8.89 ±0.1^g^	7.01 ±0.1^j^	7.51 ±0.2^i^	11.90 ±0.2^b^	11.89 ±0.1^c^
Quercetin	18.12 ±0.5^b^	21.12 ±0.7^a^	15.80 ±0.4^c^	15.84 ±0.4^c^	15.80 ±0.4^c^	nd	15.70 ±0.3^d^	15.72 ±0.3^d^	15.80 ±0.3^c^	14.45 ±0.3^e^
**Total Flavonoids**	42.67	54.65	35.74	38.96	41.90	21.83	22.71	23.23	45.60	36.87
Phenolic acids										
Vanillin	11.23 ±0.3^e^	9.41 ±0.3^i^	8.27 ±0.3^j^	10.56 ±0.2^g^	13.13 ±0.2^d^	18.01 ±0.4^a^	15.65 ±0.3^b^	10.82 ±0.2^f^	14.90 ±0.3^c^	9.63 ±0.2^h^
*p*-Hydroxybenzaldehyde	14.40 ±0.1^b^	6.08 ±0.3^f^	2.24 ±0.1^h^	8.96 ±0.4^d^	0.32 ±0.2^i^	18.82 ±0.3^a^	9.56 ±0.1^c^	4.64 ±0.2^g^	6.96 ±0.2^e^	nd
*p*-Hydroxybenzoic acid	8.48 ±0.1^c^	9.52 ±0.2^b^	7.92 ±0.2^d^	6.96 ±0.2^f^	7.60 ±0.2^e^	9.60 ±0.3^b^	9.28 ±0.2^b^	5.52 ±0.1^g^	11.36 ±0.2^a^	3.44 ±0.1^h^
*p*-Coumaric acid	nd	0.58 ±0.1^f^	0.84 ±0.1^f^	1.82 ±0.1^e^	2.80 ±0.2^d^	8.00 ±0.3^a^	5.86 ±0.2^b^	2.38 ±0.1^d^	4.98 ±0.2^c^	nd
Protocatechuic acid	3.16 ±0.3^i^	2.90 ±0.2^j^	4.46 ±0.2^h^	7.18 ±0.3^e^	9.36 ±0.2^b^	8.40 ±0.3^c^	11.64 ±0.3^a^	4.54 ±0.2^g^	7.48 ±0.1^d^	5.38 ±0.1^f^
Syringic acid	0.02 ±0.1^j^	5.52 ±0.3^e^	3.24 ±0.3^g^	3.64 ±0.3^f^	1.92 ±0.2^i^	5.88 ±0.3^d^	6.02 ±0.3^c^	9.42 ±0.3^a^	6.54 ±0.2^b^	2.26 ±0.2^h^
Ferulic acid	94.98 ±0.7^d^	114.84 ±1.3^a^	107.90 ±1.5^b^	7.04 ±0.3^j^	58.20 ±0.6^i^	77.12 ±0.6^g^	80.50 ±0.8^f^	58.96 ±0.6^h^	85.50 ±0.9^e^	111.50 ±0.9^c^
Sinapic acid	81.10 ±0.8^e^	103.50 ±1.3^a^	95.64 ±0.9^b^	42.00 ±0.4^h^	68.56 ±0.7^f^	80.18 ±0.8^e^	69.26 ±0.5^f^	49.74 ±0.5^g^	86.00 ±0.7^d^	91.00 ±0.9^c^
Vanillic acid	11.28 ±0.2^g^	15.24 ±0.3^d^	17.04 ±0.3^a^	15.32 ±0.2^c^	nd	9.58 ±0.1^i^	11.38 ±0.2^f^	16.74 ±0.2^b^	12.88 ±0.2^e^	9.74 ±0.1^h^
**Total phenolic acids**	224.65	267.59	247.55	103.48	161.89	235.59	219.15	162.76	236.6	232.95

Values are mean ± standard deviation (n = 3). Values with different letters in the same row indicate significant differences (P < 0.05). nd: not detected.

**Table 4:** Deep eutectic solvents used in this study.

DES	Composition	Molar Ratio
DES-1	Choline chloride/glycerol	1:2
DES-2	Choline chloride/1,4-butanediol	1:6
DES-3	Choline chloride/D-fructose	1:1
DES-4	Choline chloride/D-glucose	3:2
DES-5	Choline chloride/lactic acid	1:2
DES-6	Choline chloride/DL-malic acid	1:1
DES-7	Choline chloride/urea	1:2

**Table 5:** Retention times, MW, and MS and MS^2^ fragmentation ions of 9 phenolic and 3 flavonoid compounds analyzed.

Compounds	RT (min)	MW	[M−H]^−^ (m/z)			MS^2^ Fragment Ions (m/z)
			Measured	Calculated	Error (ppm)	
Protocatechuic acid	1.57	154.120	153.0190	153.0193	−2.15	91.099; 108.084; 109.179
*p*-Hydroxybenzoic acid	2.60	138.121	137.0243	137.0244	−0.59	65.238; 75.046; 93.220
Procyanidin B1	2.65	578.520	579.1480	579.1497	−2.87	289.088;407.009; 425.148
Catechin	3.20	290.268	291.0862	291.0863	−0.56	203.256; 245.129
Vanillic acid	3.38	168.147	167.0346	167.0350	−2.31	91.078; 108.118; 123.106; 152.027
*p*-Hydroxybenzaldehyde	3.76	122.121	121.0291	121.0295	−3.51	92.224; 120.306
Syringic acid	4.07	198.173	197.0447	197.0455	−3.94	95.222; 123.166; 167.117; 182.117
Vanillin	5.02	152.147	151.0398	151.0401	−1.83	51.600; 92.239; 107.962; 136.138
*p*-Coumaric acid	5.82	164.158	163.0397	163.0401	−2.39	93.221; 117.187; 119.119
Ferulic acid	6.68	194.184	193.0503	193.0506	−2.05	133.097;134.166; 167.117; 182.117
Sinapic acid	6.84	224.210	223.0603	223.0612	−4.08	149.105;164.141; 193.081; 208.100
Quercetin	8.18	302.236	303.0494	303.0499	−1.61	107.184;121.176; 151.074; 179.060

RT: Retention time; MW: molecular weight; MS: mass.

**Table 6:** Investigated variables and their levels in a three-level Box–Behnken (BBD) model.

Variable	Symbol	Coded Level		
		−1	0	1
Water contents (%w/w)	A	10	30	50
Temperature (℃)	B	30	45	60
S/L ratio^1^	C	30	50	70

^1^ wild rice powder (mg) per mL of DES.

## Supplementary Materials

The following are available online.

## References

[1] Zhai C.K., Lu C.M., Zhang X.Q., Sun G.J., Lorenz K.J. (2001). Comparative Study on Nutritional Value of Chinese and North American Wild Rice. J. Food Compos. Anal..

[2] Yan N., Du Y., Liu X., Chu C., Shi J., Zhang H., Liu Y., Zhang Z. (2018). Morphological Characteristics, Nutrients, and Bioactive Compounds of *Zizania latifolia*, and Health Benefits of Its Seeds. Molecules.

[3] Jiang M.X., Zhai L.J., Yang H., Zhai S.M., Zhai C.K. (2016). Analysis of Active Components and Proteomics of Chinese Wild Rice (*Zizania latifolia* (Griseb) Turcz) and *Indica* Rice (*Nagina22*). J. Med. Food.

[4] Sumczynski D., Kotaskova E., Orsavova J., Valasek P. (2017). Contribution of individual phenolics to antioxidant activity and in vitro digestibility of wild rices (*Zizania aquatica* L.). Food Chem..

[5] Qiu Y., Liu Q., Beta T. (2010). Antioxidant properties of commercial wild rice and analysis of soluble and insoluble phenolic acids. Food Chem..

[6] Qiu Y., Liu Q., Beta T. (2009). Antioxidant activity of commercial wild rice and identification of flavonoid compounds in active fractions. J. Agric. Food Chem..

[7] Lee S.S., Baek Y.S., Eun C.S., Yu M.H., Baek N.I., Chung D.K., Bang M.H., Yang S.A. (2015). Tricin derivatives as anti-inflammatory and anti-allergic constituents from the aerial part of *Zizania latifolia*. Biosci. Biotechnol. Biochem..

[8] Han S.F., Zhang H., Zhai C.K. (2012). Protective potentials of wild rice (*Zizania latifolia* (Griseb) Turcz) against obesity and lipotoxicity induced by a high-fat/cholesterol diet in rats. Food Chem. Toxicol..

[9] Chu M.J., Liu X.M., Yan N., Wang F.Z., Du Y.M., Zhang Z.F. (2018). Partial Purification, Identification, and Quantitation of Antioxidants from Wild Rice (*Zizania latifolia*). Molecules.

[10] Tahira R., Naeemullah M., Akbar F., Masood M. (2011). Major phenolic acids of local and exotic mint germplasm grown in islamabad. Pak. J. Bot..

[11] Russo B., Picconi F., Malandrucco I., Frontoni S. (2019). Flavonoids and Insulin-Resistance: From Molecular Evidences to Clinical Trials. Int. J. Mol. Sci..

[12] Tumova S., Kerimi A., Williamson G. (2019). Long term treatment with quercetin in contrast to the sulfate and glucuronide conjugates affects HIF1α stability and Nrf2 signaling in endothelial cells and leads to changes in glucose metabolism. Free Radic. Biol. Med..

[13] Lee C.W., Chi M.C., Chang T.M., Liu J.F. (2018). Artocarpin induces cell apoptosis in human osteosarcoma cells through endoplasmic reticulum stress and reactive oxygen species. J. Cell. Physiol..

[14] Zainal-Abidin M.H., Hayyan M., Hayyan A., Jayakumar N.S. (2017). New horizons in the extraction of bioactive compounds using deep eutectic solvents: A review. Anal. Chim. Acta.

[15] Wang M., Wang J.Q., Zhou Y.Y., Zhang M.Y., Xia Q., Bi W.T., Chen D.D.Y. (2017). Ecofriendly Mechanochemical Extraction of Bioactive Compounds from Plants with Deep Eutectic Solvents. ACS Sustain. Chem. Eng..

[16] Tan T., Zhang M., Wan Y., Qiu H. (2016). Utilization of deep eutectic solvents as novel mobile phase additives for improving the separation of bioactive quaternary alkaloids. Talanta.

[17] Shang X., Tan J.N., Du Y., Liu X., Zhang Z. (2018). Environmentally-Friendly Extraction of Flavonoids from *Cyclocarya paliurus* (Batal.) Iljinskaja Leaves with Deep Eutectic Solvents and Evaluation of Their Antioxidant Activities. Molecules.

[18] Mansur A.R., Song N.E., Jang H.W., Lim T.G., Yoo M., Nam T.G. (2019). Optimizing the ultrasound-assisted deep eutectic solvent extraction of flavonoids in common buckwheat sprouts. Food Chem..

[19] Liu W., Zong B.Y., Yu J.J., Bi Y.L. (2018). Ultrasonic-Assisted Liquid-Liquid Microextraction Based on Natural Deep Eutectic Solvent for the HPLC-UV Determination of Tert-Butylhydroquinone from Soybean Oils. Food Anal. Method.

[20] Liu W., Zhang K.D., Yu J.J., Bi Y.L. (2017). A Green Ultrasonic-Assisted Liquid-Liquid Microextraction Based on Deep Eutectic Solvent for the HPLC-UV Determination of TBHQ in Edible Oils. Food Anal. Method.

[21] Guo N., Ping K., Jiang Y.W., Wang L.T., Niu L.J., Liu Z.M., Fu Y.J. (2019). Natural deep eutectic solvents couple with integrative extraction technique as an effective approach for mulberry anthocyanin extraction. Food Chem..

[22] Fernandez M.L.A., Espino M., Gomez F.J.V., Silva M.F. (2018). Novel approaches mediated by tailor-made green solvents for the extraction of phenolic compounds from agro-food industrial by-products. Food Chem..

[23] El Kantar S., Rajha H.N., Boussetta N., Vorobiev E., Maroun R.G., Louka N. (2019). Green extraction of polyphenols from grapefruit peels using high voltage electrical discharges, deep eutectic solvents and aqueous glycerol. Food Chem..

[24] Duan L., Dou L.L., Guo L., Li P., Liu E.H. (2016). Comprehensive evaluation of deep eutectic solvents in extraction of bioactive natural products. ACS Sustain. Chem. Eng..

[25] Cvjetko Bubalo M., Curko N., Tomasevic M., Kovacevic Ganic K., Radojcic Redovnikovic I. (2016). Green extraction of grape skin phenolics by using deep eutectic solvents. Food Chem..

[26] Cao Q., Li J., Xia Y., Li W., Luo S., Ma C., Liu S. (2018). Green Extraction of Six Phenolic Compounds from Rattan (*Calamoideae faberii*) with Deep Eutectic Solvent by Homogenate-Assisted Vacuum-Cavitation Method. Molecules.

[27] Yoo D.E., Jeong K.M., Han S.Y., Kim E.M., Jin Y., Lee J. (2018). Deep eutectic solvent-based valorization of spent coffee grounds. Food Chem..

[28] Bajkacz S., Adamek J. (2017). Evaluation of new natural deep eutectic solvents for the extraction of isoflavones from soy products. Talanta.

[29] Garcia A., Rodriguez-Juan E., Rodriguez-Gutierrez G., Rios J.J., Fernandez-Bolanos J. (2016). Extraction of phenolic compounds from virgin olive oil by deep eutectic solvents (DESs). Food Chem..

[30] Huang Y., Feng F., Jiang J., Qiao Y., Wu T., Voglmeir J., Chen Z.G. (2017). Green and efficient extraction of rutin from tartary buckwheat hull by using natural deep eutectic solvents. Food Chem..

[31] Paradiso V.M., Clemente A., Summo C., Pasqualone A., Caponio F. (2016). Towards green analysis of virgin olive oil phenolic compounds: Extraction by a natural deep eutectic solvent and direct spectrophotometric detection. Food Chem..

[32] Cao J., Chen L.Y., Li M.H., Cao F.L., Zhao L.G., Su E.Z. (2018). Two-phase systems developed with hydrophilic and hydrophobic deep eutectic solvents for simultaneously extracting various bioactive compounds with different polarities. Green Chem..

[33] Zhou P.F., Wang X.P., Liu P.Z., Huang J., Wang C., Pan M., Kuang Z.S. (2018). Enhanced phenolic compounds extraction from *Morus alba* L. leaves by deep eutectic solvents combined with ultrasonic-assisted extraction. Ind. Crop. Prod.

[34] Ozturk B., Parkinson C., Gonzalez-Miquel M. (2018). Extraction of polyphenolic antioxidants from orange peel waste using deep eutectic solvents. Sep. Purif. Technol..

[35] Dai Y., van Spronsen J., Witkamp G.J., Verpoorte R., Choi Y.H. (2013). Natural deep eutectic solvents as new potential media for green technology. Anal. Chim. Acta.

[36] Rodrigues F., Palmeira-de-Oliveira A., das Neves J., Sarmento B., Amaral M.H., Oliveira M.B. (2015). Coffee silverskin: a possible valuable cosmetic ingredient. Pharm. Biol..

[37] Singleton V.L., Orthofer R., Lamuela-Raventos R.M. (1999). Analysis of total phenols and other oxidation substrates and antioxidants by means of folin-ciocalteu reagent. Method. Enzymol..

[38] Amarowicz R., Pegg R.B., Rahimi-Moghaddam P., Barl B., Weil J.A. (2004). Free-radical scavenging capacity and antioxidant activity of selected plant species from the Canadian prairies. Food Chem..

[39] Re R., Pellegrini N., Proteggente A., Pannala A., Yang M., Rice-Evans C. (1999). Antioxidant activity applying an improved ABTS radical cation decolorization assay. Free Radic. Biol. Med..

