# Supplementary material for: Optimizing Ultrasound-Assisted Deep Eutectic Solvent Extraction of Bioactive Compounds from Chinese Wild Rice
Source: Molecules. 2019 Jul 26;24(15):2718. doi: 10.3390/molecules24152718 (PMC6696331; doi:10.3390/molecules24152718)
Supplement: Supplementary file 1 [file molecules-24-02718-s001.pdf]

Supplementary Information

Optimizing Ultrasound-Assisted Deep Eutectic Solvent Extraction of Bioactive Compounds from Chinese Wild Rice

Jia Zeng [1,2,†], Yuqing Dou [1,†], Ning Yan [1], Na Li [1,2], Huaibao Zhang [1,*] and Jia-Neng Tan [1,3,*]

[1] Tobacco Research Institute of Chinese Academy of Agricultural Sciences, Qingdao 266101, China
[2] Graduate School of Chinese Academy of Agricultural Sciences, Beijing 100081, China
[3] Key Laboratory of Oilseeds Processing, Ministry of Agriculture, Wuhan 430062, China
* Correspondence: zhanghuaibao@caas.cn (H.Z.); tanjianeng@caas.cn (J.-N.T.); Tel.: +86-532-8870-2052; Fax: +86-532-8870-2239 (H.Z.)
† These authors contributed equally to this work.

Table S1

Parameters of linear regression, concentration rang, LOD and LOQ of the analyzed standards.

standards	Regression equation	Concentration rang (μg/g)	R^2	LOD (ng/mL)	LOQ (ng/mL)
Vanillin	y = 330396x + 58002.6	0.30–30	0.9972	7.073	23.278
p-Hydroxybenzaldehyde	y = 37875x + 10863.4	0.2–20	0.9992	1.493	6.563
p-Hydroxybenzoic acid	y = 195770x + 3998.1	0.2–20	0.9993	6.374	22.570
p-Coumaric acid	y = 627355x + 29881.7	0.05–5	0.9995	3.547	13.621
Protocatechuic acid	y = 32574x + 424.713	0.2-20	0.9998	1.348	5.704
Syringic acid	y = 16972x + 12950.9	0.02-10	0.9989	7.469	25.581
Ferulic acid	y = 18581.6x – 40196.1	0.5-120	0.9944	3.241	10.834
Sinapic acid	y = 222815x + 65084	0.5-120	0.9995	4.684	15.302
Vanillic acid	y = 341171x – 73698.3	0.2-20	0.9921	7.561	16.320
Catechin	y = 39552.9x – 23122.9	0.5-25	0.9998	9.747	30.422
Procyanidin B1	y = 29832.4x – 16919.9	0.5-20	0.9985	4.360	14.071
Quercetin	y = 235670x – 42503	0.5-20	0.9924	0.479	2.690

The Regression equation was constructed by plotting the peak area versus the concentration of each standard. LOD, limits of detection. LOQ, limits of quantification.

Fig. S1

The UPLC-MS/MS TIC spectrum of the standards.

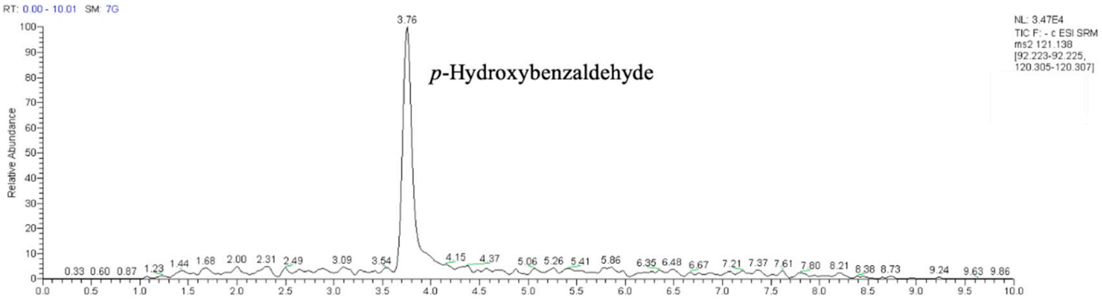
RT: 0.00 - 10.01 SM: 7G
p-Hydroxybenzaldehyde
NL: 3.47E4
TIC F: - c ESI SRM ms2 121.138 [92.223-92.225, 120.305-120.307]
Relative Abundance
3.76
0.33 0.60 0.87 1.23 1.44 1.68 2.00 2.31 2.49 3.09 3.54 4.15 4.37 5.06 5.26 5.41 5.86 6.35 6.48 6.67 7.21 7.37 7.61 7.80 8.21 8.38 8.73 9.24 9.63 9.86

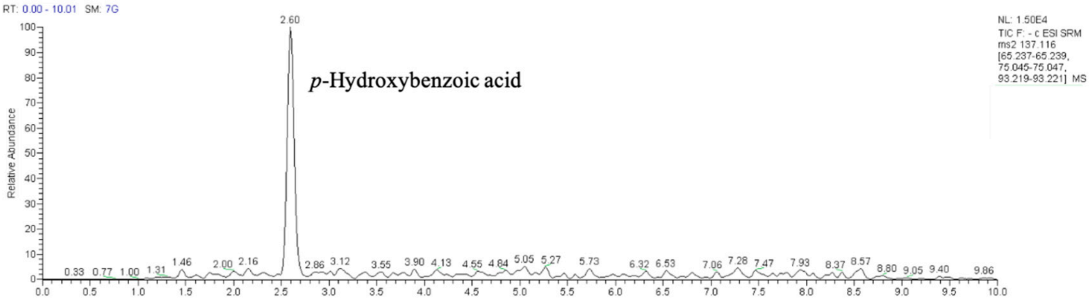
RT: 0.00 - 10.01 SM: 7G
p-Hydroxybenzoic acid
NL: 1.50E4
TIC F: - c ESI SRM ms2 137.116 [65.237-65.239, 75.045-75.047, 93.219-93.221] MS
Relative Abundance
2.60
0.33 0.77 1.00 1.31 1.46 2.00 2.16 2.86 3.12 3.55 3.90 4.13 4.55 4.84 5.05 5.27 5.73 6.32 6.53 7.06 7.28 7.47 7.93 8.37 8.57 8.80 9.05 9.40 9.86

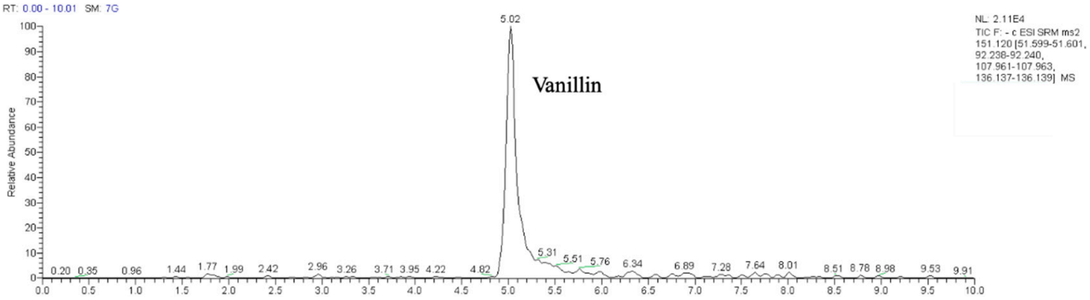
RT: 0.00 - 10.01 SM: 7G
Vanillin
NL: 2.11E4
TIC F: - c ESI SRM ms2 151.120 [51.599-51.601, 92.238-92.240, 107.961-107.963, 136.137-136.139] MS
Relative Abundance
5.02
0.20 0.35 0.96 1.44 1.77 1.99 2.42 2.96 3.26 3.71 3.95 4.22 4.82 5.31 5.51 5.76 6.34 6.89 7.28 7.64 8.01 8.51 8.78 8.98 9.53 9.91

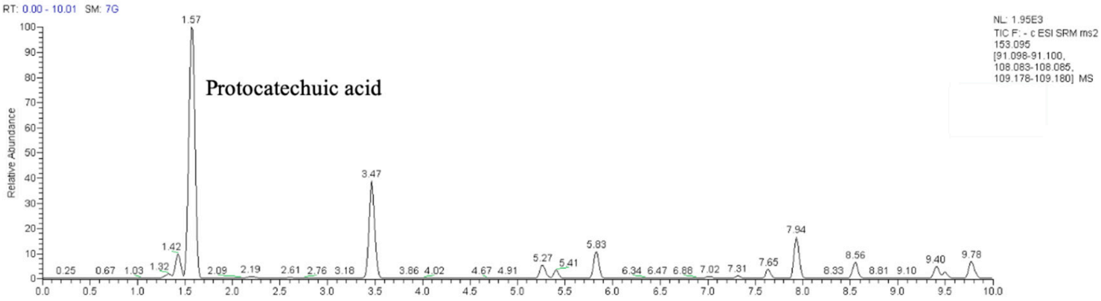
RT: 0.00 - 10.01 SM: 7G
Protocatechuic acid
NL: 1.95E3
TIC F: - c ESI SRM ms2 153.095 [91.098-91.100, 108.083-108.085, 109.178-109.180] MS
Relative Abundance
1.57
3.47
0.25 0.67 1.03 1.32 1.42 2.09 2.19 2.61 2.76 3.18 3.86 4.02 4.67 4.91 5.27 5.41 5.83 6.34 6.47 6.88 7.02 7.31 7.65 7.94 8.33 8.56 8.81 9.10 9.40 9.78

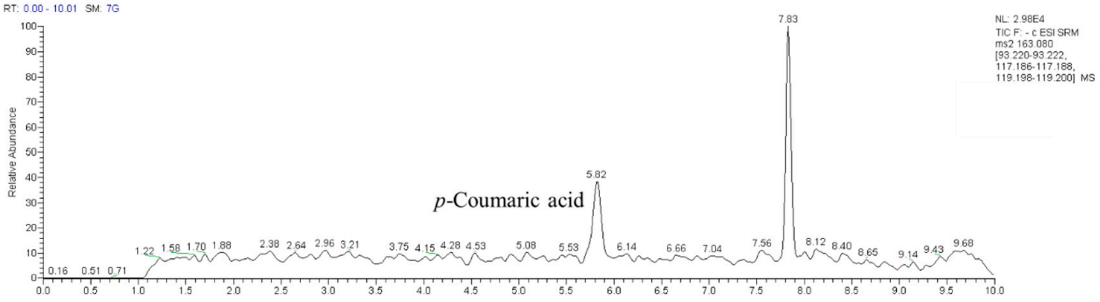
RT: 0.00 - 10.01 SM: 7G
p-Coumaric acid
NL: 2.98E4
TIC F: - c ESI SRM ms2 163.080 [93.220-93.222, 117.186-117.188, 119.198-119.200] MS
Relative Abundance
7.83
5.82
0.16 0.51 0.71 1.22 1.58 1.70 1.88 2.38 2.64 2.96 3.21 3.75 4.15 4.28 4.53 5.08 5.53 6.14 6.66 7.04 7.56 8.12 8.40 8.65 9.14 9.43 9.68

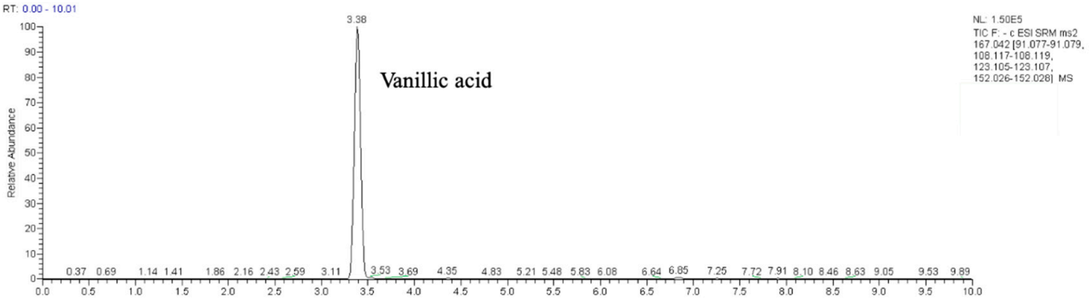
RT: 0.00 - 10.01
3.38
Vanillic acid
NL: 1.50E5
TIC F: - c ESI SRM ms2 167.042 [91.077-91.079, 108.117-108.119, 123.105-123.107, 152.026-152.028] MS
Relative Abundance

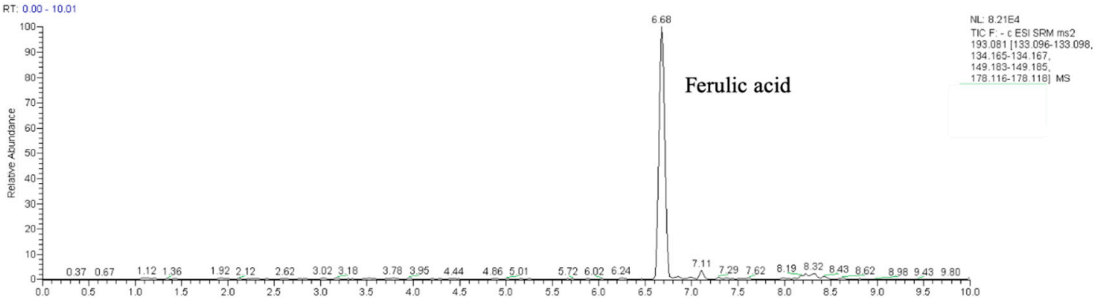
RT: 0.00 - 10.01
6.68
Ferulic acid
NL: 8.21E4
TIC F: - c ESI SRM ms2 193.081 [133.096-133.098, 134.165-134.167, 149.183-149.185, 178.116-178.118] MS
Relative Abundance

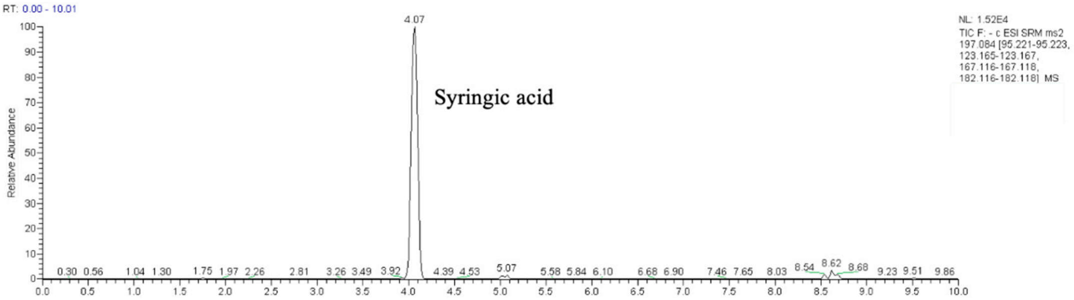
RT: 0.00 - 10.01
4.07
Syringic acid
NL: 1.52E4
TIC F: - c ESI SRM ms2 197.084 [95.221-95.223, 123.165-123.167, 167.116-167.118, 182.116-182.118] MS
Relative Abundance

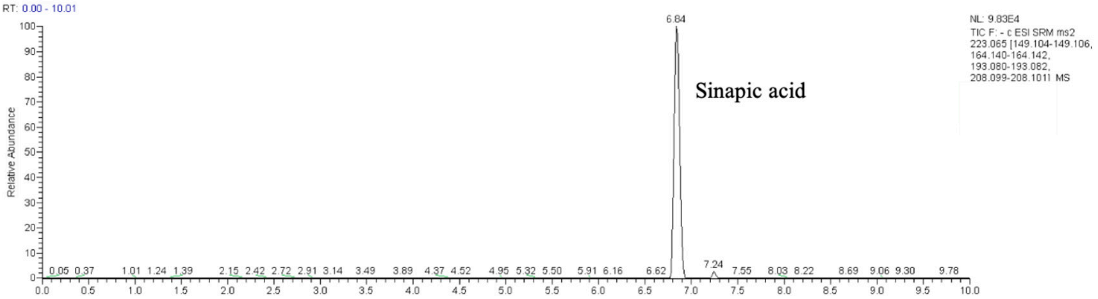
RT: 0.00 - 10.01
6.84
Sinapic acid
NL: 9.83E4
TIC F: - c ESI SRM ms2 223.065 [149.104-149.106, 164.140-164.142, 193.080-193.082, 208.099-208.101] MS
Relative Abundance

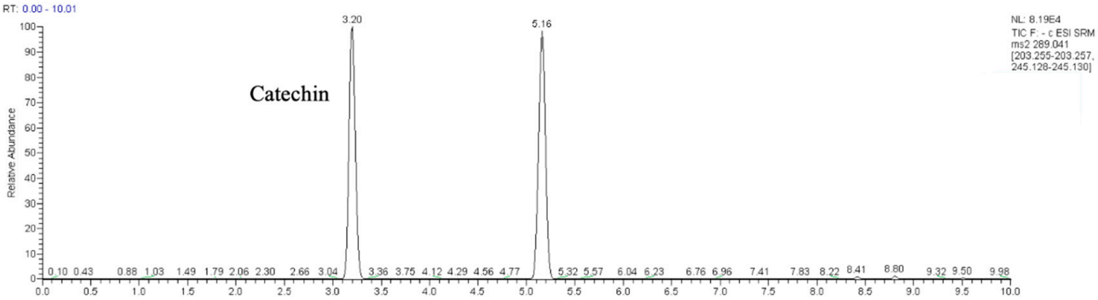
RT: 0.00 - 10.01
3.20
5.16
Catechin
NL: 8.19E4
TIC F: - c ESI SRM ms2 289.041 [203.255-203.257, 245.128-245.130]
Relative Abundance

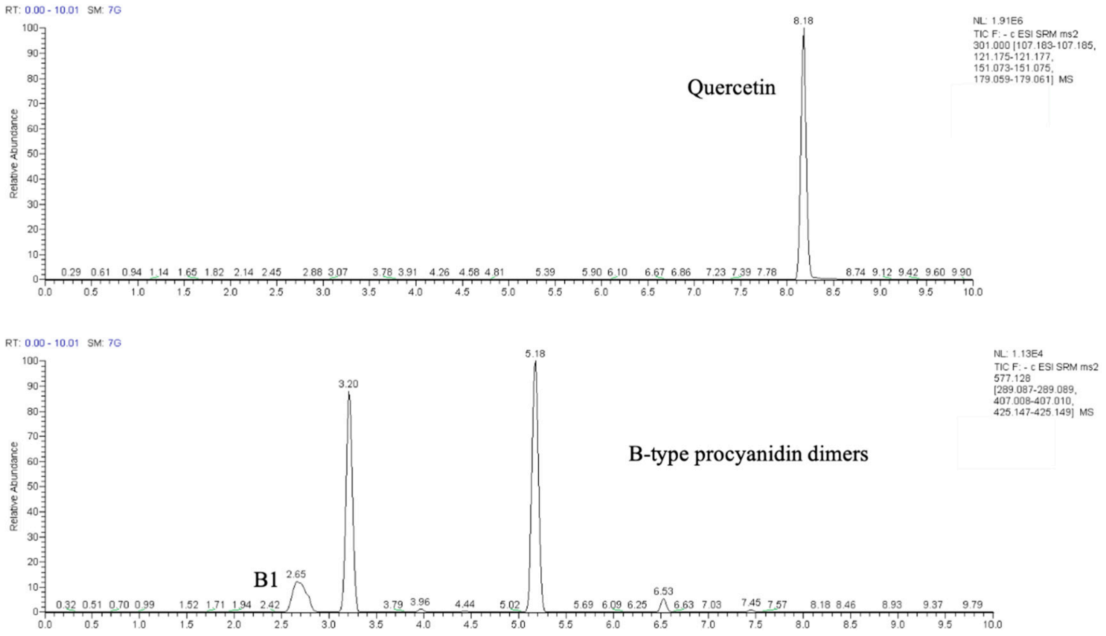

Fig.S2

Correlations between (a) TFC and DPPH assay results, (b) TFC and ABTS assay results.

(a)

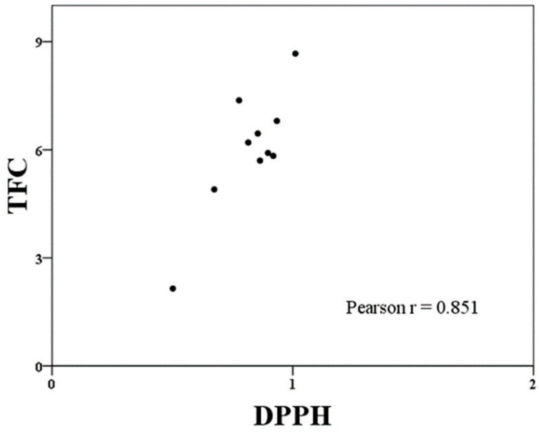

(b)

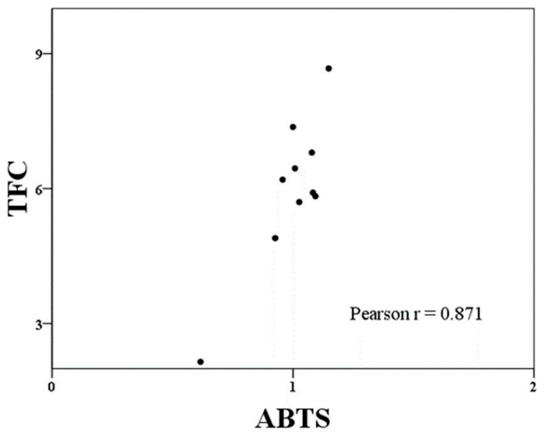
TFC
9
6
3
Pearson r = 0.871
0
1
2
ABTS
